# Electroacupuncture Reduces Cerebral Hemorrhage Injury in Rats by Improving Cerebral Iron Metabolism

**DOI:** 10.1155/2022/6943438

**Published:** 2022-08-16

**Authors:** Qiuxin Chen, Wenjing Song, Yihe Tang, Yizhou Tang, Yuying Kang, Luwen Zhu

**Affiliations:** ^1^First Affiliated Hospital of Heilongjiang University of Chinese Medicine, Harbin, China; ^2^Heilongjiang University of Chinese Medicine, China; ^3^Second Affiliated Hospital of Heilongjiang University of Chinese Medicine, Harbin, China

## Abstract

**Objective:**

To study the effects of electroacupuncture at Baihui and Dazhui points on the expression of hepcidin (Hepc), transferrin (Tf), transferrin receptor (TfR), and ferritin (Ft) in rats with cerebral hemorrhage to provide a theoretical basis for the treatment of cerebral hemorrhage with acupuncture.

**Method:**

The model of cerebral hemorrhage in rats was established by autologous blood injection method and treated by electroacupuncture (EA) at the acupoints of Baihui and Dazhui. Hepc siRNA was injected into the lateral ventricle 30 min before model preparation to produce the cerebral hemorrhage model. The modified neurological severity score (mNSS) was used to assess the neurological function, and the total iron content in brain tissue was determined using atomic absorption spectrometry; the expression of Hepc, Ft, Tf, and TfR in perihematoma tissue was detected using immunohistochemistry; the interference efficiency of Hepc siRNA was detected using western blot and reverse transcription polymerase chain reaction (RT-PCR).

**Results:**

The degree of neurological deficit showed a downward trend at 3 days, 7 days, and 14 days, and electroacupuncture significantly reduced the neurological deficit score at each time point (*P* < 0.01). Regarding total iron content in brain tissue, on the 3rd day, the 7th day, and the 14th day, the iron content of the hematoma tissue after intracerebral hemorrhage was reduced by electroacupuncture (*P* < 0.01). Regarding immunohistochemical results. Hepc, Ft, Tf, and TfR protein expressions on day 14 were significantly higher after cerebral hemorrhage (*P* < 0.01). After electroacupuncture, the expression of Hepc, Ft, Tf, and TfR protein was significantly reduced (*P* < 0.01). Western blot and RT-PCR revealed that the interference efficiency of Hepc siRNA was statistically significant (*P* < 0.01).

**Conclusion:**

Electroacupuncture can reduce neurological severity scores in rats with cerebral hemorrhage and may exert cerebral protective effects by reducing Hepc protein and gene expression; lowering Ft, Tf, and TfR protein expression; and promoting iron metabolism in the brain of rats with cerebral hemorrhage.

## 1. Introduction

Cerebral hemorrhage (ICH) is a subtype of stroke that accounts for approximately 10–20% of all strokes and has a 1-month mortality rate of 20–40%, with higher incidence, morbidity, and mortality than those of ischemic stroke [1–3]. The important factors contributing to the poor prognosis of patients are secondary injuries after cerebral hemorrhage, including apoptosis, inflammatory response, tissue necrosis, erythrocyte lysis, and cerebral edema [4, 5]. In recent years, it has been found that iron deposited in the tissue surrounding the hematoma after cerebral hemorrhage is an important factor contributing to secondary brain injury [6]. After cerebral hemorrhage, hemoglobin is broken down into iron ions and bilirubin by heme oxygenase 1 (HO-1), the rate-limiting enzyme of heme catabolism, which has strong oxidative properties and plays an important role in pathological responses such as neuronal cell injury and secondary brain edema [7, 8]. Among them, hepcidin (Hepc) is an essential iron-regulating peptide hormone that plays an important role in maintaining the balance of iron metabolism in the body and cells by regulating intestinal iron absorption, serum iron concentration, and tissue iron distribution [9, 10].

Electroacupuncture (EA) is another type of acupuncture that originated from the combination of acupuncture and electrical stimulation. Because of its relatively simple, inexpensive, and highly feasible treatment characteristics, it is generally accepted by stroke patients in clinical practice [11]. Our previous study [12] found that acupuncture improved the symptoms of neurological deficits and reduced pathological inflammatory injury in rats with cerebral hemorrhage by regulating the expression of HO-1 and inflammatory factors; however, there has been no in-depth study from the perspective of iron metabolism. In this experiment, we used electroacupuncture at the Baihui and Dazhui acupoints to treat rats with cerebral hemorrhage to observe the effect of electroacupuncture on the expression of Hepc, ferritin(Ft), transferrin(Tf), and transferrin receptor(TfR) proteins in the surrounding tissue of the hematoma. Introducing siRNA to interfere with the expression of Hepc, by constructing siRNA against the Hepc gene, the gene expression of Hepc was reduced, so as to explore the correlation between brain iron metabolism and Hepc expression after cerebral hemorrhage and the effect of electroacupuncture to explore the pathological mechanism of secondary brain injury after ICH from the perspective of iron metabolism.

## 2. Materials and Methods

### 2.1. Experimental Animals

90 healthy 8-week-old SPF-grade male Wistar rats weighing 300 ± 20 g (provided by the Animal Experiment Center, Norman Bethune Health Science Center of Jilin University, Animal License No. SCXK(Ji) 013-0004) were housed in a ventilated room in the Brain Function and Neurorehabilitation Laboratory of Heilongjiang University of Chinese Medicine, maintained at a temperature of 22 ± 3°C, relative humidity of 60 ± 5%, and 12 h/12 h alternating light/dark cycles, and there is an ultraviolet disinfection system to disinfect the animal room on a regular basis, the animal room is well ventilated, and the food and water sources are sufficient and hygienic. The experiment was approved by the Animal Care and Use Committee at the Heilongjiang University of Chinese Medicine (No. hljngnsysll202108), and experiments followed the relevant provisions of the “Guideline on Welfare of Laboratory Animals” issued by the Ministry of Science and Technology.

### 2.2. Main Reagents and Instruments

Hepc polyclonal antibody (ab30760, abcam, UK), Tf antibody, TfR antibody, Ft antibody (10727-1-AP, Proteintech, China), stereotaxic instruments (ST-5ND-C, Chengdu Instrument Factory, China), and an electric thermostatic incubator (DH36001B, Tianjin Teste, Tianjin, China) were used.

### 2.3. Model Construction

A rat model of cerebral hemorrhage was constructed by referencing the literature [13, 14]. The rats were anesthetized with intraperitoneal injection of 1% pentobarbital sodium (50 mg/kg), fixed in the prone position on the stereotaxic apparatus, and prepared for skin disinfection. A midline incision was made, and a periosteal dissector was used to dissect the periosteum and expose the fontanelle and coronal suture; 3.5 mm to the right and 0.2 mm posterior of the fontanelle point (Bregma's point) was located and a circular hole of 1.0 mm in diameter was made with a dental drill to reach the dura surface. The rat's tail was disinfected using alcohol, and the tail was cut 3 cm from the caudal end. Fifty microliters of blood was obtained using a microinjector, which was fixed on the stereotaxic apparatus, and the needle was inserted approximately 6 mm along the drill hole. Fifty microliters of nonheparinized blood was pushed into the caudate nucleus at a rate of 20 *μ*l/min, and the needle was left in place for approximately 5 min and slowly withdrawn. During needle retention, alcohol-soaked cotton balls were used to bandage the wound at the cut end of the rat's tail, gentamicin was sprayed locally after surgery, the skull wound was closed with dental cement, the scalp was sutured, and the local skin was disinfected with Iodine phenol. Animals in the sham group underwent the same surgical procedures as those in the model group, but no blood injection was performed.

Six hours after surgery was performed, the Berderson scoring method [15] was used to determine successful animal models. Rats with scores of 1 to 3 were included in the experiment. We used hematoxylin-eosin staining for pathological detection of cerebral ischemia model.

### 2.4. Grouping and Intervention Methods

**Figure 1:**
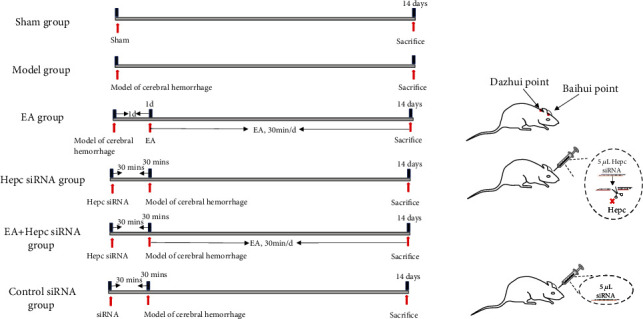
Grouping and intervention methods.

Seventy-two male Wistar rats were divided into sham, model, EA group, Hepc siRNA group, EA+Hepc siRNA, or control siRNA groups using a random number table method, with 12 rats in each group, as shown in (Figure 1). The remaining 18 rats will be used as substitutes in the event of an accident in the experiment.

Animals in the sham group received various surgical procedures similar to those performed in the treated group, but without blood injection. Normal handling and fixation were performed without any intervention.

Animals in the model group only underwent cerebral hemorrhage modeling; normal handling and fixation were performed without any intervention.

In EA group, a cerebral hemorrhage model was induced. Treatment was started on day 1 after surgery until the animals were euthanized. By referring to the “Development of Acupoint Atlas of Rats” [16], the Baihui acupoint (in the middle of the parietal bone) and Dazhui acupoint (between the seventh cervical vertebra and first thoracic vertebra in the middle of the back) were selected, and the Huatuo no. 30 0.5-inch milli-needle was used to perform acupuncture at the Baihui and Dazhui acupoints, at a depth of 10 mm and connected to a G6805-2A electroacupuncture instrument (Shanghai Huayi). Stimulation was provided with sparse and dense waves, at a frequency of 2 Hz/15 Hz, intensity of 2 mA, and duration of 30 min. Acupuncture was performed once per day.

In the Hepc siRNA group, a viral suspension of 5 *μ*l Hepc siRNA was injected into the lateral ventricle 30 min before induction of a cerebral hemorrhage model. Normal handling and fixation were performed without any intervention.

In the EA+Hepc siRNA group, 5 *μ*l of viral suspension of Hepc siRNA was injected into the lateral ventricle 30 min before induction of a cerebral hemorrhage model. The remaining steps were the same as those in the EA group.

In the control siRNA group, a viral suspension of 5 *μ*l scrambled siRNA was injected into the lateral ventricle 30 min before model construction to construct a model of cerebral hemorrhage. Normal handling and fixation were performed without any intervention.

### 2.5. Synthesis of Hepc siRNA and Lentiviral Vector Construction

A small interfering RNA fragment of Hepc (Hepc siRNA) and an ineffective small interfering RNA fragment as a control (scramble siRNA) were designed and synthesized by Wanlei Life Sciences (Shenyang, China) biological company. Plasmids were constructed and packaged with lentiviral particles and stored briefly at 4°C for subsequent experiments.

### 2.6. Observation Markers and Testing Methods

After surgery (3 d, 7 d, and 14 d), neurological deficit scoring was carried out for each group of rats. At 14 days after surgery, rats were deeply anesthetized using intraperitoneal injection of 2% pentobarbital sodium (0.4–0.5 ml/100 g). Six rats were selected from each group for intracardiac perfusion. Brain tissue was then excised from rats in each group to remove the olfactory bulb and tissue from 4 mm in front of the frontal pole, from the middle of the right cerebral hemisphere, with the injection needle tract as the center, and each sample was sliced with a coronal thickness of about 5 mm. Tissues were embedded in paraffin and stored for immunohistochemistry; six other rats were selected, brain tissue was then excised from rats in each group to remove the olfactory bulb and tissue from 4 mm in front of the frontal pole, from the middle of the right cerebral hemisphere, with the injection needle tract as the center, and each sample was sliced with a coronal thickness of about 5 mm, frozen at −80°C, and kept for subsequent total iron content assay, western blotting, and reverse transcription polymerase chain reaction (RT-PCR) assay.

### 2.7. Neurobehavioral Evaluation

The modified neurological severity score (mNSS) was used to detect neurological impairment in six groups of rats [17, 18]. The scores ranged from 0 to 18, with 1–6 representing mild impairment, 7–12 representing moderate impairment, and 13–18 representing severe impairment.

### 2.8. Determination of Total Iron Content in Perihematoma Tissue

A 0.05 g sample of brain tissue was accurately weighed, rinsed repeatedly with deionized water, dried in a 60°C oven, nitrated with high-quality pure nitric and perchloric acids, and underwent high temperature ashing until colorless or slightly yellowish transparent crystals were obtained. Its ionized water was added to 5 ml, and the total iron content in brain tissue was measured using flame atomic absorption spectrometry [19].

### 2.9. Detection of Hepc, Ft, Tf, and TfR Expressions in Perihematomal Tissue Using Immunohistochemistry

The expression of Hepc, Ft, Tf, and TfR in the perihematomal tissue was detected through brain tissue embedding, sectioning, clearing and hydration, antigen retrieval, hydrogen peroxide incubation, goat serum blocking, primary antibody incubation, secondary antibody incubation, horseradish enzyme labeling, DAB color development, hematoxylin counterstaining, dehydration, clearing, mounting, and microscopic examination. The mean optical densities of proteins were measured.

### 2.10. Western Blot Measurement of Interference Efficiency of Hepc siRNA in Perihematoma Tissues

After total protein extraction, the protein concentration was determined using the BCA method, followed by resolution with SDS-PAGE at the corresponding concentration, transfer to membranes, addition of the corresponding primary and secondary antibodies for immunoreactivity, and addition of ECL luminescent solution for exposure. The relative expression of the target protein and *β*-actin was calculated by detecting the grayscale value (OD) of the bands using Quantity One analysis software (BIO-RAD company USA).

### 2.11. RT-PCR Measurement of Interference Efficiency of Hepc siRNA in Perihematoma Tissues

Rat brain tissues were collected, and total RNA was extracted by adding TRIzol. cDNA was obtained through reverse transcription of total RNA and used as the sample for detection of the *β*-actin and target genes. A housekeeping gene was used for normalization, and the primers shown in Table 1 were provided by Wanlei Life Sciences Co., Ltd. (Shenyang, China). Relative expression analysis of the above markers was performed using the 2-*ΔΔ*Ct method.

### 2.12. Statistical Processing

The data were statistically analyzed using SPSS22.0. Normally distributed quantitative data are expressed as mean ± standard deviation ($\bar{x}\pm s$), and one-way analysis of variance was used to compare between groups at each time point. The least significant difference *t*-test was used for pairwise comparison. A difference with *P* < 0.05 was considered statistically significant.

## 3. Results

### 3.1. Comparison of Neurofunctional Behavioral mNSS Scores between Groups' Pathological Results of Cerebral Hemorrhage Model

**Figure 2:**
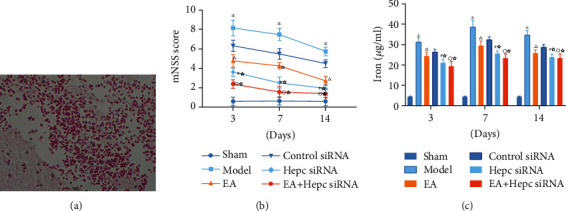
mNSS scores and total iron content of rats in each group. Note: ^∗^*P* < 0.01 vs. sham group, ^△^*P* < 0.01 vs. model group; ^☆^*P* < 0.01 vs. EA group, ^#^*P* < 0.01 vs. control siRNA group, and ^○^*P* < 0.01 vs. Hepc siRNA group.

Pathological results of cerebral hemorrhage model are shown in Figure 2(a). All rats had an mNSS score of 0 before scoring. The sham group had no significant postoperative neurological deficits. The rats in the model group showed significant signs of neurological deficit by day 3 and still showed signs of neurological deficit on days 7 and 14. Compared with the model group, the mNSS scores of rats in the electroacupuncture group were significantly lower at all time points (*P* < 0.01). Compared with the EA group, the mNSS scores of rats in the Hepc siRNA and EA+Hepc siRNA groups were significantly lower at all time points (*P* < 0.01). Compared with the Hepc siRNA group, the neurological function scores of rats in the EA+Hepc siRNA group were significantly lower at all time points (*P* < 0.01) (Figure 2(b)).

### 3.2. Comparison of Total Iron Content of Rats in Each Group

Rats in the sham group had a small amount of iron in the brain after surgery. The iron content in the brains of rats in the model group was significantly higher on days 3, 7, and 14. Compared with the model group, the iron content in the brains of rats in the EA group was significantly lower at all time points (*P* < 0.01). Compared with the electroacupuncture group, the iron content in the brains of rats in the Hepc siRNA and EA+Hepc siRNA groups at all time points was significantly lower (*P* < 0.01). Compared with the Hepc siRNA group, the EA+Hepc siRNA group had significantly lower iron content in rat brain at all time points (*P* < 0.01) (Figure 2(c)).

### 3.3. Immunohistochemical Results of Hepc, Ft, Tf, and TfR in Perihematoma Tissue

**Figure 3:**
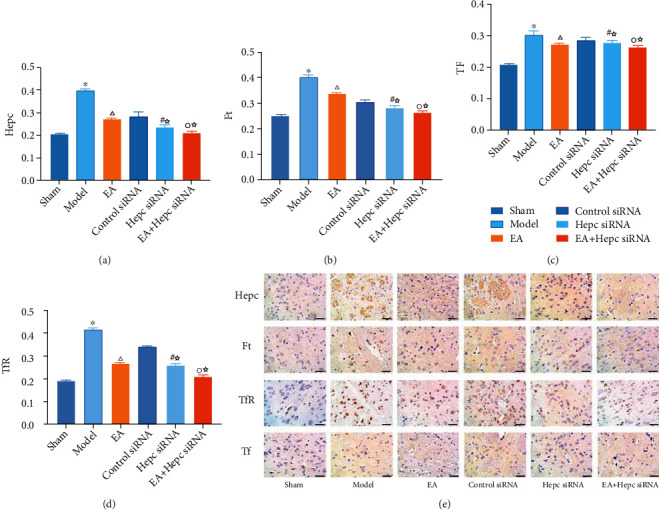
Immunohistochemical results of Hepc and Ft, Tf, and TfR in each group. Note: ^∗^*P* < 0.01 vs. sham group, ^△^*P* < 0.01 vs. model group, ^☆^*P* < 0.01 vs. EA group, ^#^*P* < 0.01 vs. control siRNA group, and ^○^*P* < 0.01 vs. Hepc siRNA group.

On day 14 after surgery, weak positive expression of Hepc, Ft, Tf, and TfR was seen in the sham group. Expression of Hepc, Ft, Tf, and TfR protein was significantly higher in the model group (*P* < 0.01). Compared with that in the model group, Hepc, Ft, Tf, and TfR protein expression was lower in the EA group (*P* < 0.01). Compared with that in the EA group, Hepc, Ft, Tf, and TfR protein expression was lower in the Hepc siRNA and EA+Hepc siRNA groups (*P* < 0.01). Compared with that in the Hepc siRNA group, Hepc, Ft, Tf, and TfR protein expression was lower in the EA+Hepc siRNA group (*P* < 0.01) (Figure 3).

### 3.4. Western Blotting Results of Hepc Protein in Perihematoma Tissue

**Figure 4:**
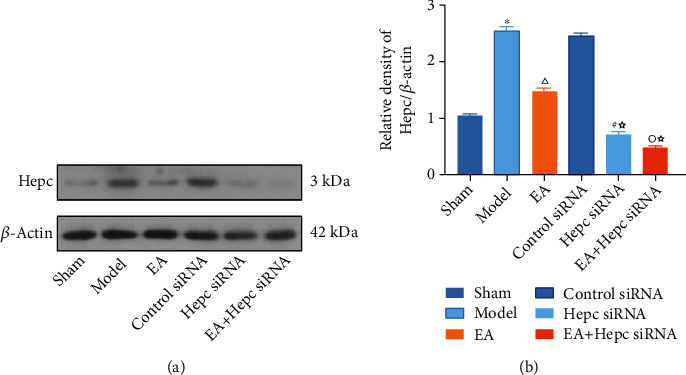
Western blotting results of Hepc protein. Note: ^∗^*P* < 0.01 vs. sham group, ^△^*P* < 0.01 vs. model group, ^☆^*P* < 0.01 vs. EA group, ^#^*P* < 0.01 vs. control siRNA group, and ^○^*P* < 0.01 vs. Hepc siRNA group.

On day 14 after surgery, weak positive expression of Hepc was seen in the sham group. Hepc protein expression was significantly higher in the model group (*P* < 0.01). Compared with that in the model group, Hepc protein expression was lower in the EA group (*P* < 0.01). Compared with that in the EA group, Hepc protein expression was lower in the Hepc siRNA and EA+Hepc siRNA groups (*P* < 0.01). Compared with that in the Hepc siRNA group, Hepc protein expression was lower in the EA+Hepc siRNA group (*P* < 0.01) (Figure 4).

### 3.5. RT-PCR Results of Hepc mRNA in Perihematomal Perihematoma Tissue

**Figure 5:**
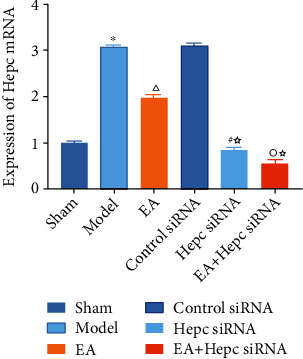
RT-PCR results of Hepc mRNA.

Weak positive expression of Hepc mRNA was seen in the sham group on day 14 after surgery. Hepc mRNA expression was significantly higher in the model group (*P* < 0.01). Compared with that in the model group, Hepc mRNA expression was lower in the EA group (*P* < 0.01). Compared with that in the EA group, Hepc mRNA expression was lower in the Hepc siRNA and EA+Hepc siRNA groups (*P* < 0.01). Compared with that in the Hepc siRNA group, Hepc mRNA expression was lower in the EA+Hepc siRNA group (*P* < 0.01) (Figure 5).

## 4. Discussion

Neurological deterioration in patients with cerebral hemorrhage is attributed to several modifiable factors, including acute hematoma growth, intracerebroventricular dilatation, and perihematoma edema [20]. After cerebral hemorrhage, iron ions released by erythrocyte lysis in the hematoma play a key role in mediating secondary neuronal injury and edema formation [4, 21]. Iron levels in brain tissue surrounding the lesion remain significantly higher than normal during recovery, and the deposited iron can be reactivated to cause delayed brain injury, although the pathogenesis is unclear [22, 23]. Hemoglobin released by the disintegration of red blood cells in the blood can be decomposed in the presence of HO-1 to produce Fe^2+^, CO, and biliverdin, resulting in iron overload in the brain, where the nonheme iron content can increase by threefold; the main mechanisms of neurodegeneration in hemorrhagic stroke are iron and heme-induced ROS production, amplification of inflammatory responses, direct toxic effects of iron and heme, and glutamate-induced excitotoxicity [24, 25]. As a result, damaged mitochondria produce many reactive oxygen species [26]. The resulting disruption of glutamine metabolism leads to dysfunction of antioxidant pathways and hydroxyl radical synthesis, which in turn leads to neuronal ferroptosis [27–29]. Iron ions are involved in the metabolic processes of brain cells, and low or high levels of iron ions in brain tissue can cause functional brain damage [7]. The amount of iron deposition in the brain tissue of rats after cerebral hemorrhage gradually increases in the brain injury area, pathological damage of brain tissue gradually increases, and the amount of iron deposition positively correlates with the neurological severity score of rats, suggesting that excessive iron deposition in brain tissue is an important factor leading to brain injury [30]. The imbalance of iron metabolism in patients after cerebral hemorrhage and elevated serum iron-regulating factor levels are associated with poorer prognosis in patients, and the trend of elevation is consistent with the severity of neurological deficit and prognosis of patients [31].

After cerebral hemorrhage, iron metabolism in the brain is imbalanced and the body activates the Fe-transferrin -TfR pathway to maintain its own homeostasis, promoting the efflux of iron ions while converting them into Ft for storage. When excess free iron exceeds the saturation capacity of Tf in the brain and generates large amounts of oxygen radicals through the Fenton reaction, it induces inflammatory responses and apoptosis [32, 33]. Wang et al. [21] demonstrated that iron overload after ICH caused oxidative damage to brain tissue, damage to the blood-brain barrier, microglial activation, and production of large amounts of tumor necrosis factor-*α* (TNF-*α*) and interleukin-1*β* (IL-1*β*), which aggravate brain edema and neuronal death. The aforementioned studies suggest that ameliorating the inflammatory response induced by the imbalance of iron metabolism in the brain is one of the key targets for the treatment of cerebral hemorrhage. Most evidence suggests that Tf/TfR1 pathway is the main route of iron transport through the luminal membrane of capillary endothelial cells [34, 35], possibly via the ferroportin1/hephaestin and/or ferroportin1/ceruloplasmin export system to transport iron as Fe^2+^ across the isolated membrane into the brain parenchyma [36–38].

Therefore, how to effectively regulate iron deposition around the hematoma in patients with cerebral hemorrhage has been a hot topic in cerebral hemorrhage research in recent years. Hepc is a newly discovered important iron-regulating hormone, which is a key substance in regulating iron homeostasis and iron metabolism in human body and plays an important role in maintaining the balance of iron metabolism in the body and cells, including hereditary hemochromatosis protein, Tfr2, and hemojuvelin [39]. The inflammatory response, hypoxic activity, erythropoietic activity, and changes in iron level that occur in the body of patients after cerebral hemorrhage lead to an increase in serum Hepc levels [40]. Hepc plays an important role in the clinical treatment of patients with cerebral hemorrhage by maintaining the balance of iron metabolism in the body through the regulation of serum iron concentration, the regulation of intestinal iron absorption, and the regulation of tissue iron distribution [41]. Clinical studies have found that serum Hepc was higher in patients with cerebral hemorrhage with poor prognosis at 1, 3, 5, and 7 d than in patients in the good prognosis group (*P* < 0.05), suggesting that serum Hepc levels in patients after cerebral hemorrhage are related to the prognostic outcome of patients [42]. Current studies on Hepc have shown different results, which may be related to the differential expression of Hepc in different parts of the brain [43]. At the same time, the production of Hepc is regulated by iron levels. In pathological states, increased circulating iron leads to upregulation of Hepc expression through feedback regulatory mechanisms, and Hepc forms complexes with membrane iron transport proteins, which induce ubiquitination of membrane iron transport proteins and negatively regulate the reduction of intracellular iron output [44, 45]. Therefore, Hepc, as a key factor of iron metabolism, plays an important regulatory role in many ways and is a new target and prognostic marker for the treatment of cerebral hemorrhage in clinical settings. Based on the above studies, in the present study, we investigated the changes in Hepc, Tf, TfR, and Ft in order to explore the mechanism of cerebral hemorrhage cases from the perspective of iron metabolism.

In the book “Compendium of Acupuncture and Moxibustion” by Yang Jizhou of the Ming Dynasty, it is stated that a three-edged needle should be used for those with acute stroke and 12 well points should be used for acupuncture. For slanted mouth and eyes, numbness of the hands and feet, and confusion, the Dazhui, Fengchi, Quchi, Zusanli, and Baihui acupoints should be used in acupuncture. For yin syndrome stroke, supplementation should be performed first followed by dissipation, with acupuncture performed on the contralateral side first and then the ipsilateral side. Among the above acupoints, Baihui is the Du meridian, which is located at the intersection of the line connecting the tips of the ears and the midline of the top of the head, where the three yang and five meetings are located, and the depth of which is where the brain is located. The Dazhui point is located in the depression below the spinous process of the 7th cervical vertebra and is where the Yang-heat Qi from the three Yang of the hands and feet converges into the Du meridian. This allows the Yang-heat qi to travel up through this point to the top of the head along the Du meridian. It can be seen that acupuncture at Baihui and Dazhui points can internally open the Du meridian and externally; they can flow through the three yang [46]. Therefore, Baihui and Dazhui points are the key points for stroke and improvement of brain function [47]. Dong et al. [48] used head acupuncture to treat the acute phase of cerebral hemorrhage, selecting head acupoints and applied acupuncture to the area from Baihui to the Taiyang acupoint to treat acute cerebral hemorrhage, breaking the paradigm that head acupuncture is prohibited in acute cerebral hemorrhagic stroke. So the Baihui and Dazhui acupoints were chosen to treat ICH rats in this study. Acupuncture can effectively promote the recovery of limb function and activities of daily living in patients with cerebral hemorrhage, and the earlier the acupuncture intervention is provided, the better the outcome [49, 50]. Electroacupuncture is a therapy in which a very slow current close to that of natural human bioelectricity is passed through the needles to treat a disease, which can cause excitation in corresponding cortical areas, enhance brain electrical activity, stimulate sensory nerve impulses, induce the corresponding part of brain cells to awaken, and restore brain function. Several studies have shown that electroacupuncture can reduce the volume of cerebral edema in patients; improve their neurological, vascular, and cognitive abilities; and improve their activities of daily living and quality of life [51–55]. And studies have confirmed that acupuncture can improve neuronal ferroptosis after cerebral hemorrhage, thereby reducing the degree of nerve damage [56, 57].

In this experiment, the mNSS score results revealed that the model group rats showed different degrees of neurological deficit. The neurological scores of rats in the model group rats were highest on day 3 after surgery, and the neurological deficit symptoms of the rats gradually decreased from day 7 after surgery. Some neurological deficits were still present on day 14 after surgery, suggesting that the cerebral hemorrhage model was successful with neurological deficit symptoms. On days 3, 7, and 14, the neurological function scores were significantly lower in the electroacupuncture group than in the model group (*P* < 0.01), indicating that electroacupuncture could effectively reduce neurological function scores in rats with cerebral hemorrhage. At the same time points, the neurological function scores of the Hepc siRNA group were lower compared than those of the model group and electroacupuncture groups (*P* < 0.01), indicating that silencing the *Hepc* gene could also reduce neurological deficits in rats with cerebral hemorrhage. The EA+Hepc siRNA group had the lowest neurological function score and the most significant improvement in neurological deficit relative to the model, EA, and Hepc siRNA groups (*P* < 0.01). This shows that electroacupuncture at the Baihui and Dazhui acupoints can effectively reduce the neurological function score and improve the symptoms of neurological deficits in rats with cerebral hemorrhage. The total iron content in the brain showed the same trend as the neurological score, suggesting that the total iron content in the brain of rats with hemorrhage was directly proportional to the neurological deficit degree (*P* < 0.01) The immunohistochemistry, western blot, and RT-PCR results also reflected that electroacupuncture decreased Hepc protein and gene expression and decreased Ft, Tf, and TfR protein expressions (*P* < 0.01).

After cerebral hemorrhage rats have increased intracerebral iron content and obvious symptoms of neurological deficits, we hypothesized that electroacupuncture may promote intracerebral iron metabolism and reduce symptoms of neurological deficits in rats with cerebral hemorrhage by decreasing Hepc protein and gene expression and decreasing Ft, Tf, and TfR protein expressions. However, this study also has some limitations; although we confirmed the therapeutic effect of electroacupuncture in intracerebral iron metabolism after cerebral hemorrhage, we only based on animal experiments and could not cover the disease of the situation, so the results of this study should be used in the context of the specific situation; in addition, we only studied the intracerebral iron metabolism in the acute phase of cerebral hemorrhage and did not explore the hyperacute phase. Future research directions will include exploring the limitations of this study and conducting a more in-depth study of its mechanism of action.

## Figures and Tables

**Table 1:** Primer sequences.

Gene name	Primer sequence (5′→3′)	Product length
Hepc siRNA	Upstream: GCTGCCTGTCTCCTGCTT	159 bp
	Downstream: GGTGTCTCGCTTCCTTCG	

## Data Availability

All data used to support the findings of this study are available from the corresponding author upon reasonable request.
